# Perceived Job Insecurity and Anxiety. A Multilevel Analysis on Male and Female Workers in European Countries

**DOI:** 10.3389/fsoc.2020.573549

**Published:** 2020-09-17

**Authors:** Anna Bracci, Egidio Riva

**Affiliations:** ^1^Department of Business Economics, Health and Social Care, University of Applied Sciences and Arts of Southern Switzerland, Manno, Switzerland; ^2^Department of Sociology and Social Research, University of Milano-Bicocca, Milan, Italy

**Keywords:** anxiety, job insecurity, European countries, multilevel analysis, gender differences

## Abstract

A growing body of evidence has been produced on the adverse effects of job insecurity on health and well-being. Current research in the field conveys a few concerns, some of which are related to gender and cross-national differences in the experience of job insecurity. In order to fill these gaps this study draws on three waves (2005, 2010, 2015) of the European Working Conditions Survey and investigates, using mixed-effects logistic regression: (i) the relationship between anxiety and perceived job insecurity; and whether such relationship: (ii) is different for men and women; (iii) and varies across European countries. Results indicate that male and female workers perceiving the risk of involuntary job loss have similar odds of reporting anxiety. Furthermore, the variance in slopes across countries, relative to the general trend, is significant but modest, too, thus suggesting that the relationship under investigation is fairly similar across European countries. Implications of the findings for future research and practice are discussed.

## Introduction

Over the last few years a combination of factors, such as globalization, the widespread digitalization, labor market deregulation and the prevalence of employment in service industries over manufacturing—has made new forms of work organization more pervasive (Greenan et al., 2017). In particular, different types of non-standard or atypical employment (namely fixed-term, temporary or casual contracts) have increased and have lowered employment stability and overall social protection (Sengenberger, 2011; De Stefano, 2016). Workers in permanent contracts have also been exposed to these phenomena, which have resulted in a substantial lessening of restrictions on dismissal, as well as organizational downsizing, merges and acquisitions (OECD, 2014). Accordingly, the overall number of workers experiencing job insecurity, which is generally defined as the perceived threat of job loss and job discontinuity (De Witte et al., 2016), has widely enlarged.

A growing body of literature has proved a negative association between perceived job insecurity, on the one hand, and health or subjective well-being, effective functioning at work, and occupational outcomes, on the other hand (for meta-analyses and literature reviews, see e.g., Sverke et al., 2002; Cheng and Chan, 2008; Berkman et al., 2014; De Witte et al., 2016). Accordingly, the issue of job insecurity has been relatively high on the policy-making agenda and it has gained increasing attention in a wide range of disciplines. Nonetheless, current research in the field conveys a few concerns, some of which are related to gender and cross-national differences in the experience of job insecurity (László et al., 2010).

The constant and rapid growth in female employment rates has surely expanded the opportunities women may benefit from; however, it is widely demonstrated that female workers tend to have less favorable work and employment conditions compared to their male counterparts (Felstead et al., 2015; Grimshaw et al., 2017). In particular, although several studies indicate that women usually report better working time quality and face fewer physical hazards, there is evidence of severe and persistent gender inequalities in careers patterns and contractual arrangements (Fagan and Burchell, 2002; Olsen et al., 2010; Green and Mostafa, 2012; Eurofound, 2017a). Indeed, across the world, women are highly concentrated in specific sectors and occupations and are overrepresented in temporary or non-standard employment (ILO, 2016), partly as a result of the greater difficulties they face in entering and remaining into the labor market due to the unequal distribution of time allocated to unpaid work. That said, findings on gender-specific consequences of precarious jobs on health and well-being are not conclusive. Some studies found that the strength of the association between precariousness and health is fairly similar among men and women (Pelfrene et al., 2003; Green, 2011; Reichert and Tauchmann, 2017; Kachi et al., 2018; Menéndez-Espina et al., 2019). Other research suggested that responses to adverse work and employment conditions may be different for men and women (Kalil et al., 2010; Cottini, 2012). Finally, a number of studies indicate that the link between job insecurity and health among female workers is negligible (Wang et al., 2008; Watson and Osberg, 2017).

Furthermore, to the best of our knowledge, there is little published data on cross-national differences in the relationship between the fear of involuntary job loss and health or well-being. Economic and political variables (such as labor market regulation, employment protection legislation, welfare policies, unemployment rate) as well as prevailing cultural frameworks could predict diversity in health-related outcomes of the experience of job insecurity (Erlinghagen, 2008; László et al., 2010; Balz, 2017). Nonetheless, most research in the field has so far investigated single countries, such as UK (Ferrie et al., 2005), Germany (Reichert and Tauchmann, 2017), Spain (Menéndez-Espina et al., 2019), Finland (Griep et al., 2016), Denmark (Cottini and Ghinetti, 2018), the United States (Simmons and Swanberg, 2008), Japan (Kachi et al., 2018), Australia (Green, 2011), and Canada (Wang et al., 2008). Even the studies of Buffel et al. (2015) and Caroli and Godard (2016), whose analyses drew on large and international datasets, have not provide cross-country comparative analyses. Hence, there is abundant room to advance the understanding of how perceived job insecurity may differently impact the well-being and health of (female) workers in different countries.

In order to contribute filling these gaps, this study draws on the European Working Conditions Survey (EWCS) (Eurofound, 2017b)—which is a nationally representative dataset of employees and the self-employed in European countries—and investigates, using multilevel modeling: (i) the association between perceived job insecurity and a self-reported measure of anxiety from a gender perspective; (ii) whether such relationship varies across countries. The topic is quite important for policy purposes, given that labor market change, along with welfare retrenchment, has produced new social risks (Bonoli, 2005), which have mainly affected women (OECD, 2017). In addition, psychological well-being is crucial for both employers and national governments, as long as it has a wide range of direct and indirect social and economic costs (OECD, 2012). For instance, in the public health framework, policymakers should pay specific attention to women employed in insecure jobs, since women are more exposed to the medicalization of social problems and more likely to use medical treatment as a common coping mechanism (Buffel et al., 2015).

The remainder of this paper is structured as follows. The next section begins by laying out the theoretical background and literature review. Section Data and Methods is concerned with the data and the methodology used for this study. The fourth section presents the findings. Section Results discusses the results, while the final section discusses some implications for policy-making and future research.

## Literature Review and Research Propositions

Previous research indicates that perceived job insecurity is a stressor, which is therefore positively correlated to poorer mental well-being, in general, and anxiety, in particular (Simmons and Swanberg, 2008; Burgard et al., 2009; Milner et al., 2016; Cottini and Ghinetti, 2018; Wood and Burchell, 2018). For instance, Simmons and Swanberg (2008) found that job insecurity is the single most significant correlate of depressive symptoms for working poor employees. Some findings also prove that the fear of involuntary job loss is comparable to unemployment in affecting mental health (Green, 2011; Griep et al., 2016; Reichert and Tauchmann, 2017).

The most authoritative frameworks on the health and behavioral consequences of job insecurity, such as work stress theories, highlight that social or psychological variables act as key determinants or moderator variables (Sverke et al., 2002; De Witte et al., 2016). The perceived risk of involuntary job loss is expected to have adverse effects due to a stressful work environment, which does not provide enough rewards in return for the high efforts (as in the effort-reward imbalance model, see Siegrist and Wahrendorf, 2016), or due to a lack of control that workers may have over their jobs (as in the job demand-control model, see Karasek, 1979). More in general, experiences, such as the anticipation of frustration at work, unpredictability and uncontrollability, the consumption of available resources (Janlert and Hammarström, 2009), which are all associated to the prolonged uncertainty inherent in job insecurity, may lead to reduced mental health and well-being.

Job insecurity may produce health-related negative outcomes as a consequence of gender-specific employment patterns and exposure to different work and employment conditions. We mainly refer to the fact that female workers, compared to their male counterparts, tend to be segregated into passive or low-control jobs, which are associated with poorer health status (O'Campo et al., 2004; Cottini, 2012), or report lower overall job quality (Simões et al., 2015). Furthermore, on average, women still fulfill the large majority of unpaid work obligations. Hence employment, especially when insecure, may have adverse effects on health also because of possible work-family conflicts (Menéndez et al., 2007; Wang et al., 2008; Avendano and Berkman, 2014). In this respect, literature highlights that a variety of coping strategies and social support may buffer the detrimental consequences that the experience of job insecurity could have on job attitudes and health among women (see e.g., Kalil et al., 2010; Menéndez-Espina et al., 2019). Hence, based on previous literature, we may anticipate the following hypothesis:

H1: Perceived job insecurity predicts anxiety, in both the female and male subsamples

Turning to cross-country differences, distinct reactions to precariousness may arise from peculiar country-level variables and characteristics. In countries where breadwinner norms are still predominant, the association between job insecurity and health may be comparatively lower for women compared to men, as these are expected to contribute the most to the household income. In addition, the non-economic gender approach, which attributes inequalities to patriarchy and gender stereotypes within institutions and interactional contexts, could explain the low or negligible effects of insecurity as a stressor among women (Wang et al., 2008; Cloutier et al., 2009; Stier and Yaish, 2014). On the other hand, within a more gender-egalitarian culture, the negative effects of perceived job insecurity may be significant for women as much as for men (Kachi et al., 2018). Within this general framework, the results of the study conducted by László et al. (2010), one of the few that have adopted a cross-country perspective, are quite interesting. Using cross-sectional data, the authors have analyzed the association between job insecurity and health among working people aged 45–70 years in 16 European countries and have obtained similar results in both male and female subsamples. In more detail, this study shows that job insecurity is significantly associated with an increased risk of poor health in countries, such as Czech Republic, Denmark, Germany, Greece, Hungary, Israel, the Netherlands, Poland, and Russia; conversely, small or no relationships are found in Austria, France, Italy, Spain and Switzerland, Belgium and Sweden. It is quite evident that the grouping of countries does not seem to follow any of the existing models in the welfare regimes or varieties of capitalism literature (e.g., Esping-Andersen, 1990; Hall and Soskice, 2001) or in cross-cultural studies (e.g., Hofstede, 1991). Based on these findings we may reason that:

H2: Male and female workers perceiving the risk of involuntary job loss have similar odds of reporting anxiety

*H3: The relationship between job insecurity and anxiety varies across countries*.

## Data and Methods

### Sample

This study employs three waves (2005, 2010, 2015) of the European Working Conditions Survey (EWCS) (Eurofound, 2017b). Due to the geographical coverage of the survey in all three waves, the original sample was initially restricted to 30 European countries (the 28 EU member states plus Norway and Turkey) and subsequently further reduced to include only those who answered the question assessing the dependent variable (*N* = 90,048). Both male (*N* = 45,621) and female (*N* = 44,427) and male workers were predominantly aged 15–44 years (55.6 and 54.6%, respectively) and well-educated: 26.6% of sampled men and 34.6% of their female counterparts have achieved tertiary qualifications. The large majority of men (79.5%) are employees and work in the private sector (79.6%), mainly in manufacturing (18.3%), other services (14.0%), wholesale and retail trade (13.8%), and construction activities (12.3%). Sampled women are comparatively more present in the public sector (35.4%) and predominantly work in other services (40.3%), wholesale or retail trade (16.0%), and manufacturing (11.2%). Finally, it is worth mentioning that 46.8% of female workers are the person who contributes the most to the household income. For comprehensive descriptive statistics, see Table 1.

**Table 1:** Summary statistics, by gender.

	**Men**		**Women**	
	***N*.**	**Freq. (%)**	***N*.**	**Freq. (%)**
**AGE**				
15/34	13,474	29.7	12,266	27.7
35/44	11,795	26.0	11,889	26.9
45+	20,174	44.4	20,093	45.4
**EDUCATION**				
Primary	3,399	7.7	2,264	5.2
Secondary	29,147	65.7	26,055	60.2
Tertiary	11,800	26.6	14,982	34.6
**EMPLOYMENT STATUS**				
Employee	35,608	79.5	38,235	88.1
Self-employed	9,197	20.5	5,187	11.9
**SECTOR**				
Private	33,647	79.6	26,566	65.6
Public	8,642	20.4	13,921	34.4
**WORK-LIFE FIT**				
Very well	12,146	26.8	13,945	31.6
Well	23,113	51.1	22,408	50.7
Not very well	7,605	16.8	6,321	14.3
Not at all well	2,410	53.2	1,516	34.3
**JOB SATISFACTION**				
Not at all satisfied	1,834	4.1	1,470	37.0
Not very satisfied	6,846	15.1	6,571	15.0
Satisfied	26,307	58.1	25,506	58.0
Very satisfied	10,285	22.7	10,512	23.3
**PRIMARY EARNER IN THE HOUSEHOLD**				
No	8,629	19.0	23,447	53.2
Yes	36,704	81.0	20,648	46.8

### Variables

A self-reported indicator of anxiety (“Over the last 12 months, did you have any of the following health problems? Anxiety”), dummy coded, was selected as the outcome variable of this study. The main predictor (i.e., perceived job insecurity) was measured through this question: “To what extent do you agree or disagree with following statement about your job: I might lose my job in the next 6 months.” Based on Chung (2018), the original response categories were dichotomized. In more detail, those who answered “strongly agree” or “tend to agree” were considered as perceiving the risk of involuntary job loss, whereas those who answered “neither agree or disagree,” “tend to disagree,” and “strongly disagree” were considered as those who did not feel their job insecure. Based on previous studies (Watson and Osberg, 2017; Kachi et al., 2018), a wide range of control variables were included in the model. Firstly, we added individual variables: age, recoded in three categories (15–34; 35–44; 45–54 years); educational attainment (primary, secondary, and tertiary level); employment status (employee vs. self-employed); sector (public vs. private) the respondent worked in; perceived work-life fit, ranging from 1 = very well to 4 = not very well; job satisfaction, ranging from 1 = very satisfied to 4 = not at all satisfied. In line with Caroli and Godard (2016), this latter variable was added to control for working conditions and psychosocial work environment; indeed, omitting an evaluation of working conditions could lead to an upward bias in estimations. Year of the survey and whether the respondent was the person who contributed the most to the household income were inserted as control, too. All observations with a missing value in any of the variables in the model were excluded from the analyses.

### Methods

In dealing with hierarchically structured data, we estimated a mixed-effects logistic regression model in which observations at level 1 (i.e., individuals) were nested within European countries (level 2). The model was designed with a random intercept and a random slope for perceived job insecurity in order to test whether the relationship under scrutiny might vary across countries. The variance-covariance structure of these two random effects was treated as unstructured. To make the interpretation of the regression model more meaningful, the estimated coefficients were displayed as odds ratios. Separate regression models were run for male and female subsamples.

## Results

### Descriptive Statistics

**Figure 1:**
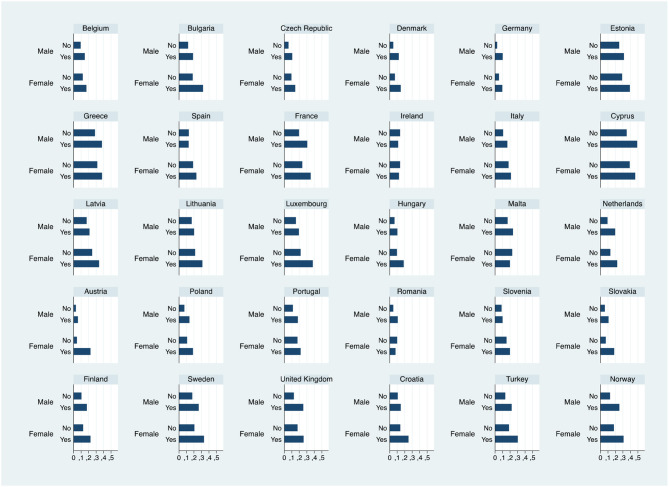
Proportion of workers who report anxiety, by job insecurity perception, gender, and country.

The observed proportion reporting anxiety across European countries is 17.8% among working women and 13.4% among working men. In Germany, Austria, Denmark, Romania, and Slovakia <10% of sampled individuals suffer from this specific health problem; on the other hand, in Estonia, Greece, and Cyprus over a third of both male and female workers report this mental problem. On average, about a quarter (24.7%) of female respondents who feel their job is insecure suffer from anxiety, whereas only 16.4% of those who do not perceive the risk of involuntary job loss are anxious. As for working men, about a fifth (18.8%) of those perceiving their job as insecure report anxiety; on the contrary, 12.4% of those who have secure jobs struggle with anxiety. As Figure 1 shows, the gap in the proportion of female workers reporting anxiety between those who perceive their job as insecure and those who do not is comparatively higher in countries, such as Austria (about 18% points), Luxembourg (about 16% points), Bulgaria (about 14% points) and lower in most Mediterranean countries (e.g., Malta, Italy, Portugal, and Spain) as well as in several continental European countries (e.g., Germany, Slovenia, and Belgium). In the male subsample such gap is relatively bigger in Cyprus (14% points), UK (12.6% points), and Norway (12.5% points) and smaller in countries, such as Ireland, Spain, Slovenia, Austria, and Lithuania.

### Mixed-Effect Logistic Regression

Results of multilevel analysis for working women are displayed in Table 2. The empty or null model (Model 1), which has no explanatory variables and allows intercepts to vary across countries, provides the decomposition of the variance of the dependent variable. The intraclass correlation coefficient is equal to 0.0855, which means that about 8.55% of the variance in the probability to report anxiety is attributable to the contextual level (i.e., level 2). Thus, a multilevel model is appropriate.

**Table 2:** Results of mixed-effects logistic regression model (female subsample).

	**Model 1**			**Model 2**			**Model 3**		
	**Odds**	**95% CI**		**Odds**	**95% CI**		**Odds**	**95% CI**	
**PERCEIVED JOB INSECURITY (NO REF. CAT.)**									
Yes				1.340^***^	1.247	1.440	1.366^***^	1.226	1.522
**AGE (15–34 REF. CAT.)**									
35/44				1.157^***^	1.069	1.252	1.154^***^	1.067	1.249
45+				1.194^***^	1.108	1.286	1.194^***^	1.108	1.286
**EDUCATION (PRIMARY REF. CAT.)**									
Secondary				0.975	0.846	1.124	0.977	0.847	1.126
Tertiary				1.212^*^	1.046	1.404	1.215^**^	1.049	1.407
**EMPLOYMENT STATUS (EMPLOYEE REF. CAT.)**									
Self-employed				1.178^**^	1.067	1.300	1.178^**^	1.067	1.300
**SECTOR (PRIVATE REF. CAT.)**									
Public sector				1.216^***^	1.139	1.299	1.216^***^	1.139	1.299
**WORK-LIFE FIT (VERY WELL REF. CAT)**									
Well				1.103^**^	1.027	1.186	1.104^**^	1.027	1.186
Not very well				1.549^***^	1.415	1.697	1.550^***^	1.415	1.697
Not at all well				2.045^***^	1.775	2.358	2.045^***^	1.774	2.357
**JOB SATISFACTION (VERY SATISFIED REF. CAT.)**									
Satisfied				1.576^***^	1.449	1.714	1.579^***^	1.452	1.717
Not very satisfied				3.159^***^	2.856	3.495	3.162^***^	2.859	3.498
Not at all satisfied				5.546^***^	4.769	6.451	5.545^***^	4.766	6.450
**PRIMARY EARNER IN THE HOUSEHOLD**									
No				0.807^***^	0.761	0.856	0.807^***^	0.761	0.855
**SURVEY WAVE**									
2010−5th EWCS				0.434^***^	0.399	0.472	0.433^***^	0.398	0.472
2015−6th EWCS				0.692^***^	0.637	0.751	0.692^***^	0.637	0.751
Constant	0.201	0.165	0.246	0.130^***^	0.099	0.171	0.129	0.098	0.171
Var (Constant)	0.308	0.183	0.516	0.316	0.187	0.534	0.334	0.197	0.565
Var (Slope)							0.043	0.012	0.151
Covar (Cons, Slope)							−0.049	−0.122	0.023
Log likelihood				−14,999.87			−14,996.34		
AIC				30,055.74			30,032.67		
*N*	44,427			35,012			35,012		

*** *p < 0.001*;

** *p < 0.01*;

* *p < 0.05*.

In Model 2 (random intercept and fixed slope model) explanatory variables at level 1 were added. Coefficients indicate that female workers who feel their job is insecure are significantly more likely to report anxiety. In particular, if a working woman reports job insecurity, her odds of suffering from anxiety are 1.34 times greater than a woman who does not perceive the risk of involuntary job loss, holding all other variables constant. As for control variables, results indicate that older females, working women with tertiary education, the self-employed and women working in the public sector, those with a poor work-life fit, those less satisfied about their job, and female workers who are the main earner in the household are more likely to report anxiety.

We run the same model on the male subsample (Table 3) and found that 11.2% of the variance in the dependent variable may be attributed to country-level variables. The findings suggest that if a worker indicates his fear of losing his job in the future, the odds of suffering from anxiety are 1,43 higher than a man who does not report the worry of future job loss, holding all other variables constant. Thus, Hypothesis 1 is confirmed. We tested possible gender-differences in the association between anxiety and perceived job insecurity by adding an interaction term in the equation: parameter estimates for gender were not significant. Thus, Hypothesis 2 is confirmed, too: there are no gender differences in the association between perceived job insecurity and anxiety.

**Table 3:** Results of mixed-effects logistic regression model (male subsample).

	**Model 1**			**Model 2**			**Model 3**		
	**Odds**	**95% CI**		**Odds**	**95% CI**		**Odds**	**95% CI**	
**PERCEIVED JOB INSECURITY (NO REF. CAT.)**									
Yes				1.427^***^	1.322	1.542	1.461^***^	1.320	1.617
**AGE (15–34 REF. CAT.)**									
35/44				1.198^***^	1.098	1.306	1.198^***^	1.099	1.306
45+				1.200^***^	1.106	1.301	1.199^***^	1.106	1.300
**EDUCATION (PRIMARY REF. CAT.)**									
Secondary				0.921	0.813	1.044	0.922	0.813	1.045
Tertiary				1.310^***^	1.144	1.496	1.308^**^	1.143	1.496
**EMPLOYMENT STATUS (EMPLOYEE REF. CAT.)**									
Self-employed				1.310^***^	1.205	1.425	1.311^**^	1.205	1.425
**SECTOR (PRIVATE REF. CAT.)**									
Public sector				1.353^***^	1.249	1.466	1.352^***^	1.247	1.465
**WORK-LIFE FIT (VERY WELL REF. CAT)**									
Well				1.022	0.939	1.112	1.022	0.939	1.112
Not very well				1.585^***^	1.435	1.751	1.583^***^	1.433	1.749
Not at all well				2.034^***^	1.783	2.321	2.033^***^	1.782	2.320
**JOB SATISFACTION (VERY SATISFIED REF. CAT.)**									
Satisfied				1.427^***^	1.300	1.567	1.425^***^	1.298	1.565
Not very satisfied				2.739^***^	2.449	3.063	2.735^***^	2.446	3.059
Not at all satisfied				4.419^***^	3.799	5.140	4.418^***^	3.797	5.139
**PRIMARY EARNER IN THE HOUSEHOLD**									
Yes				1.026	0.944	1.116	1.026	0.943	1.116
**SURVEY WAVE**									
2010−5th EWCS				0.392^***^	0.358	0.429	0.393^***^	0.359	0.431
2015−6th EWCS				0.706^***^	0.647	0.771	0.708^***^	0.648	0.772
Constant	0.136	0.070	0.175	0.083^***^	0.062	0.111	0.082^***^	0.061	0.111
Var (Constant)	0.415	0.247	0.697	0.408	0.241	0.689	0.425	0.250	0.721
Var (Slope)							0.023	0.004	0.117
Covar (Cons, Slope)							−0.035	−0.109	0.038
Log likelihood				−12,951.34			−12,949.83		
AIC				25,938.68			25,939.66		
*N*	45,621			36,392			36,392		

*** *p < 0.001*;

** *p < 0.01*;

* *p < 0.05*.

As a further step in the model building process, both intercepts and slopes were allowed to vary across countries (Model 3). Based on the parameter and standard error estimates, we may conclude that the relationship between anxiety and perceived job insecurity, which is positive and statistically significant, varies across countries for both male and female workers; however, the variance in slopes across countries is small. The covariance between intercepts and slopes is negative but not statistically significant. Post-estimation statistics, namely likelihood-ratio test and Akaike's information criteria, indicate that Model 3 fits the data better than Model 2.

**Figure 2:**
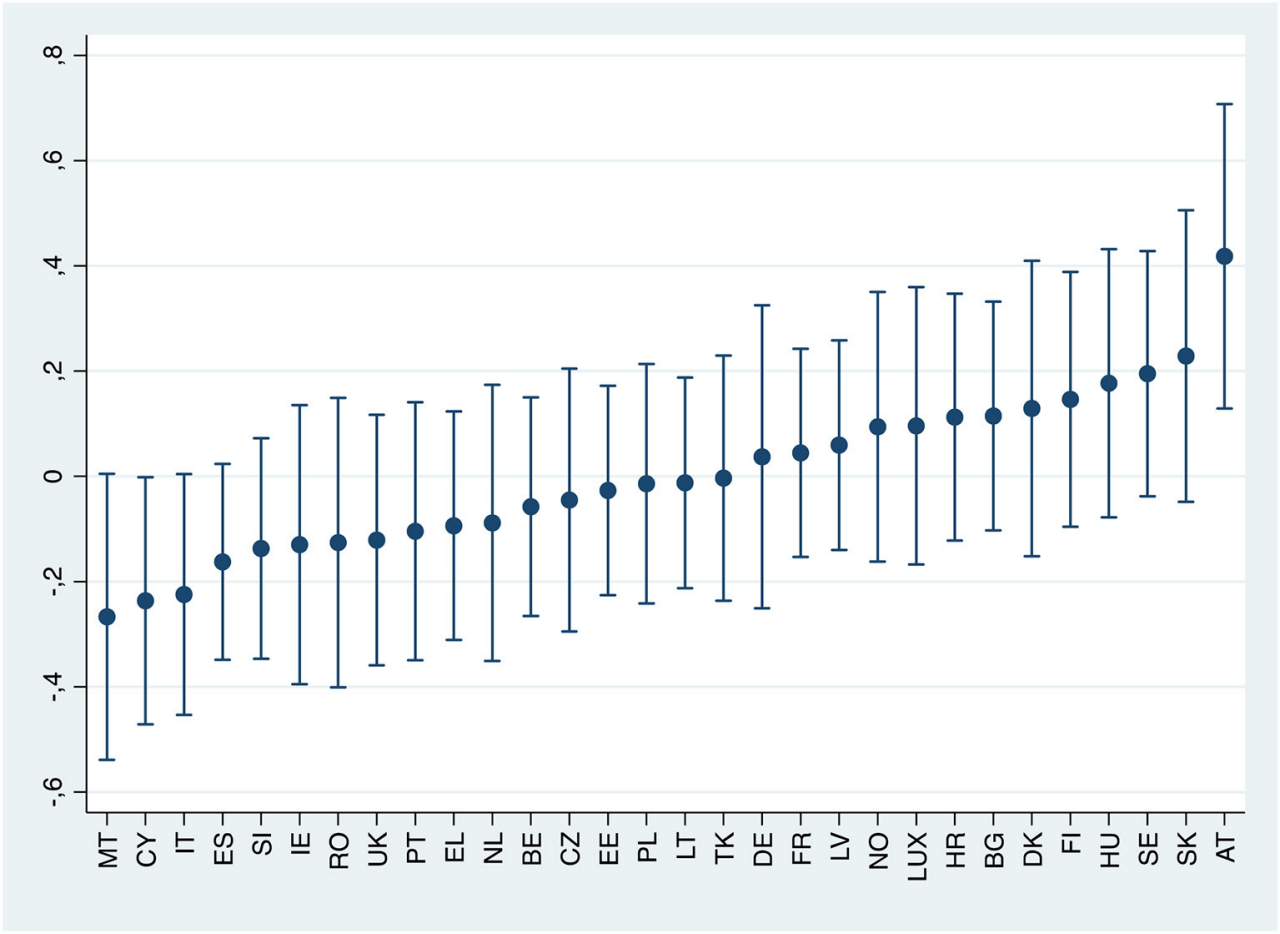
Best linear unbiased predictions of the random effects for perceived job insecurity, by country (female subsample).

**Figure 3:**
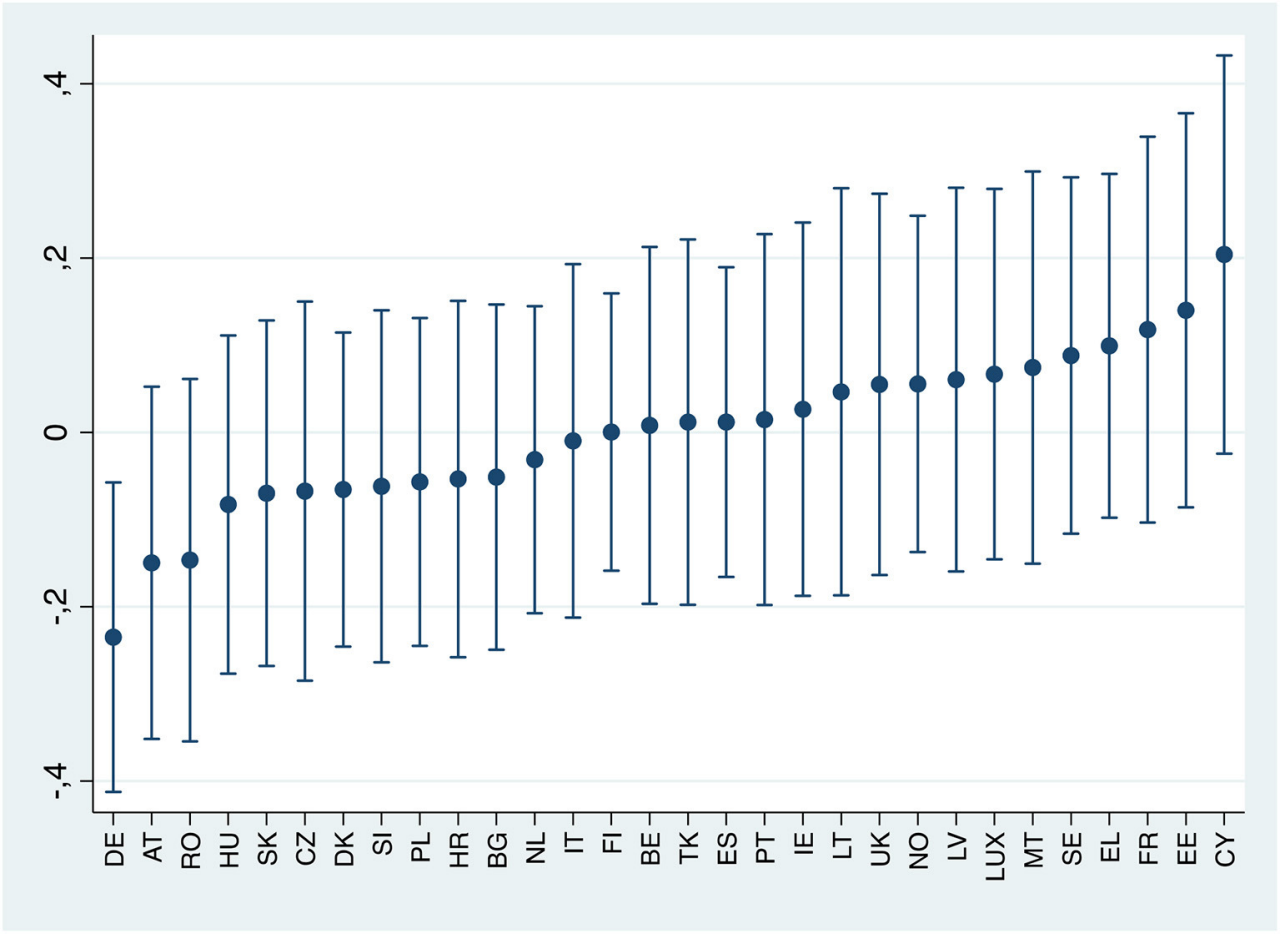
Best linear unbiased predictions of the random effects for perceived job insecurity, by country (male subsample).

Figure 2 displays the extent to which the slope of the relationship under investigation changes across countries, relative to the general trend, in the female subsample. In most countries, the probability of female workers suffering from anxiety as perceived job insecurity increases does not differ significantly from the overall pattern. Moreover, most confidence intervals of parameter estimate overlap. That means that the association between anxiety and job insecurity perception seems fairly stable across most European countries. However, it is worth noticing that the observed association is higher in Austria and lower in Cyprus, Malta and Italy, compared to the European average. Concerning men (Figure 3), the effect of perceived job insecurity on anxiety is smaller and significantly different from the European average in Germany.

## Discussion and Conclusion

The findings of this study, which prove a significant association between perceived job insecurity and anxiety in the European labor force, confirm research in the field documenting that women seem to react negatively, and in the same way as men do, to adverse work and employment conditions (Pelfrene et al., 2003; László et al., 2010; Buffel et al., 2015; Reichert and Tauchmann, 2017; Menéndez-Espina et al., 2019). The magnitude of the association between job security and anxiety may be regarded as moderate and such result is also consistent with previous evidence (László et al., 2010; Buffel et al., 2015). However, the meta-analysis conducted by Sverke et al. (2002) indicated that the fear of involuntary job loss is more strongly associated to mental health as compared to physical health; accordingly, public health consequences of job insecurity need to be seriously considered, given that the current labor market transformations (OECD, 2014) are likely to result in a higher prevalence of workers, both males and females, feeling threatened by threat of job loss.

As a second step, we employed multilevel modeling to investigate whether job insecurity could be a more pronounced stressor in specific European countries. In this regard, as in previous studies as (Erlinghagen, 2008; László et al., 2010; Balz, 2017) we found evidence of cross-national differences in the relationship between anxiety and perceived threats of job loss. However, analyses also indicate that the variation in effect sizes across countries is relatively small. Little heterogeneity in the association between job insecurity and anxiety across European countries for female workers, i.e., the main focus of this special issue, may be explained by the increasing prevalence of similar gender norms and roles and by recent trends toward greater gender egalitarianism (Kachi et al., 2018). On the other hand, some authors suggest that the risk of poor health resulting from insecure jobs may exist also in countries with good welfare regimes (Erlinghagen, 2008; László et al., 2010). We may also argue that the effects of job insecurity on mental health among working women may be triggered more by observed (and unobserved) individual and firm characteristics than contextual factors on the macro level. In this regard, our study indicates that much of the variance is attributable to variance in the intercepts (i.e., in the average levels of female workers reporting anxiety) and to micro or meso-level variables. These speculations are not mutually exclusive. In contrast to the “role theory” (Wang et al., 2008), more gender egalitarianism (i.e., beliefs about appropriate roles for men/women) seems to emerge in Europe, following major changes in women's educational attainment and employment patterns (OECD, 2019). However, as Mühlau (2011) maintains, gender-egalitarian norms do not necessarily lead to better working conditions for women. Women may need to work harder to compensate pay disadvantage, particularly in insecure and temporary jobs (Kachi et al., 2018), or to try and balance work and family responsibilities, which is still quite hard also within dual-earner and dual-career households (OECD, 2017).

As already anticipated, the findings of this study have practical implications for policymakers, as “policies that influence the length, continuity and nature of employment are likely to impact health” (Avendano and Berkman, 2014). Firstly, employment protection policies may incentive workers and employers' investments in training and safety exploiting changes in legislation to reduce insecurity from contractual arrangements. Because of the reduction of labor market rigidities, employers currently have few incentives to invest in temporary or casual workers' human capital than workers in permanent contracts. Most importantly, the nature of work should be taken into account in the field of labor policy that, in the current workfare priority, aims exclusively at a quick placement into a job (Munoz de Bustillo et al., 2011). This is particularly relevant in the context of aging workforce, where active aging policies stress the need to adapt professional conditions to older workers (Greenan et al., 2013). From a gender perspective, the predominant role that women still have as primary caregivers has strong consequences in female career and professional choices or opportunities. Tackling gender inequalities should be a key policy priority in order to provide all workers with adequate opportunities for self-validation, self-development and meeting their material needs over the life course (Grimshaw et al., 2017). In this regard, family support policies (e.g., maternity leave policies, better work-life balance arrangements, policies aimed at closing the gender wage gap) may lead to better women's health in the long run by improving their careers and earning trajectories (Avendano and Berkman, 2014; OECD, 2017).

Even if this study certainly adds to our understanding of health-related consequences of perceived job insecurity, the findings are subject to certain limitations, which need to be acknowledged. A source of weakness, which is common to most previous research, is the measurement of the dependent variable, which is a self-reported evaluation of mental health. Coherently, further research should employ objective measures and/or more comprehensive or multidimensional indicators of self-perceived of health (see e.g., Buffel et al., 2015; Moscone et al., 2016). This study was also limited by the absence of longitudinal information, which could enable the measurement of within-sample change over time and the assessment of cause-and-effect relationships, based on established sequences of events. Unfortunately, the European Working Conditions Survey, which has been used in this article, does not allow disentangling negative health risks from changes in job security and accumulated economic insecurity experiences, to whom women are particularly vulnerable (Avendano and Berkman, 2014; Watson and Osberg, 2017). Coherently, a natural progression of this study could be to address the same research question by using cross-country panel data, which could provide more definitive further evidence. De Witte et al. (2016) have systematically reviewed longitudinal studies on the association between job insecurity and health and well-being, but they have limited their examination to specific contexts. Further research in a life course perspective, which is being a predominant framework of sociologic and epidemiological research (Siegrist and Wahrendorf, 2016), can likely be extended by exploiting retrospective or longitudinal data. The longitudinal design has potentially the strength to control for unobserved selection effects, which arise when workers at high risk of accepting less secure jobs may also have certain personality characteristics (Mel Bartley and Montgomery, 2005). Caution is, hence, required to interpret and generalize our results due to the cross-sectional design, which cannot include random measurement errors due to endogeneity from omitted variables and reverse causality as well. The interaction between work and health is likely bidirectional and we cannot distinguish between social causation (bad jobs are damaging to workers' health) and direct selection (workers with poor health are more likely to be less productive and be employed in insecure jobs (De Cuyper et al., 2008). Finally, there may be an issue of self-selection into employment as job insecurity is only observed among those who work. However, a selection effect is more substantial in countries where women are more likely than men to exit the labor force because of health and family reasons, resulting in a misrepresentation of economically insecure women in the analyses (Cottini, 2012; Watson and Osberg, 2017).

## Data Availability Statement

Publicly available datasets were analyzed in this study. Info on data availability can be found here: https://www.eurofound.europa.eu/surveys/about-eurofound-surveys/data-availability#datasets.

## Author Contributions

AB has provided the theoretical support and drafted literature review. ER was responsible for data acquisition, data analysis, and presentation of the results. Both authors were responsible for discussing the results.

## Conflict of Interest

The authors declare that the research was conducted in the absence of any commercial or financial relationships that could be construed as a potential conflict of interest.
